# The Superantigen Toxic Shock Syndrome Toxin 1 Alters Human Aortic Endothelial Cell Function

**DOI:** 10.1128/IAI.00848-17

**Published:** 2018-02-20

**Authors:** Katarina Kulhankova, Kyle J. Kinney, Jessica M. Stach, Françoise A. Gourronc, Isabella M. Grumbach, Aloysius J. Klingelhutz, Wilmara Salgado-Pabón

**Affiliations:** a Department of Microbiology and Immunology, University of Iowa Carver College of Medicine, Iowa City, Iowa, USA; b Department of Internal Medicine, University of Iowa Carver College of Medicine, Iowa City, Iowa, USA; New York University School of Medicine

**Keywords:** TSST-1, human endothelial cells, superantigen, infective endocarditis, aortic endothelial cell dysfunction

## Abstract

Staphylococcus aureus infective endocarditis (IE) is a fast-progressing and tissue-destructive infection of the cardiac endothelium. The superantigens (SAgs) toxic shock syndrome toxin 1 (TSST-1), staphylococcal enterotoxin C (SEC), and the toxins encoded by the enterotoxin gene cluster (*egc*) play a novel and essential role in the etiology of S. aureus IE. Recent studies indicate that SAgs act at the infection site to cause tissue pathology and promote vegetation growth. The underlying mechanism of SAg involvement has not been clearly defined. In SAg-mediated responses, immune cell priming is considered a primary triggering event leading to endothelial cell activation and altered function. Utilizing immortalized human aortic endothelial cells (iHAECs), we demonstrated that TSST-1 directly activates iHAECs, as documented by upregulation of vascular and intercellular adhesion molecules (VCAM-1 and ICAM-1). TSST-1-mediated activation results in increased monolayer permeability and defects in vascular reendothelialization. Yet stimulation of iHAECs with TSST-1 fails to induce interleukin-8 (IL-8) and IL-6 production. Furthermore, simultaneous stimulation of iHAECs with TSST-1 and lipopolysaccharide (LPS) inhibits LPS-mediated IL-8 and IL-6 secretion, even after pretreatment with either of the proinflammatory cytokines tumor necrosis factor alpha (TNF-α) and IL-1β. IL-8 suppression is not mediated by TSST-1 binding to its canonical receptor major histocompatibility complex class II (MHC-II), supporting current evidence for a nonhematopoietic interacting site on SAgs. Together, the data suggest that TSST-1 differentially regulates cell-bound and secreted markers of endothelial cell activation that may result in dysregulated innate immune responses during S. aureus IE. Endothelial changes resulting from the action of SAgs can therefore directly contribute to the aggressive nature of S. aureus IE and development of life-threatening complications.

## INTRODUCTION

Infective endocarditis (IE) is an infection of the cardiac endothelium characterized by the formation of aggregates on heart valves consisting of host factors and bacteria, collectively called vegetations (1). Staphylococcus aureus IE is the most aggressive, tissue-destructive, and lethal form of IE, accounting for ∼40,000 cases with a 20 to 65% mortality rate annually in the United States (1–3). While there are considerable advances in general critical care management of patients with S. aureus IE, mortality remains exceedingly high (3–5). Treatment of S. aureus IE is challenging, requiring prolonged antibiotic therapy and/or surgery to replace affected valves. Infections with methicillin-resistant S. aureus are frequent, have a complicated course and treatment, and have higher mortality rates, especially in immunocompromised individuals, in whom S. aureus IE frequently leads to death (1, 2, 4). Host risk factors associated with IE have been identified. However, little is known about the S. aureus mechanisms that lead to such an aggressive form of disease. Understanding the pathophysiology of S. aureus IE is critical, given that its incidence, severity, and lethality have not decreased in the last 50 years.

S. aureus IE vegetations develop preferentially on cardiac endothelium that exhibits signs of endothelial injury, activation, and/or inflammation (6). Bacteria accumulating at the site of vascular wall injury produce numerous secreted virulence factors that aid in colonization. It has generally been accepted that S. aureus's defining mechanism for IE pathogenesis is its exceptional ability to survive in blood and adhere to endothelial tissue. Yet studies demonstrate that these pathogenic mechanisms, while essential, are not sufficient to cause acute IE (7). Recent evidence indicates that the superantigens (SAgs) toxic shock syndrome toxin 1 (TSST-1), staphylococcal enterotoxin C (SEC), and the toxins encoded by the enterotoxin gene cluster (*egc*) play a novel and essential role in the etiology of S. aureus IE (8, 9). In a rabbit model of IE, S. aureus strains with deletions of SAgs were deficient in the ability to form vegetations on heart valves. Consistent with epidemiological studies, S. aureus clinical isolates that do not encode SEC, TSST-1, and the *egc* SAgs are deficient in vegetation formation. Treatment with a soluble, high-affinity T cell receptor (TCR) Vβ protein specific to SEC and intravenous immunoglobulin (IVIG) blocks vegetation growth and abrogates lethality in experimental IE (9, 10). Superantigenicity ensues from SAg cross-linking of the TCR Vβ chain on T lymphocytes to the major histocompatibility complex class II (MHC-II) receptor on antigen-presenting cells, resulting in polyclonal CD4^+^ and CD8^+^ T cell activation and proliferation (11). The potent immune stimulating properties of SAgs can lead to systemic inflammatory syndromes, toxic shock syndrome (TSS), or septic shock (11). Of importance, superantigenicity also results from cross-linking of Vβ-TCR and MHC-II expressed on vascular endothelial cells with ICAM-1 and E-selectin serving as coreceptors, thus implicating the vascular endothelium in mediating or amplifying TSS (12–14). While adaptive immune system activation is characteristic of SAgs, this is not their only biological function. For example, TSST-1 directly induces expression of proinflammatory genes in adipocytes and vaginal epithelial cells (15–18). SAg interactions with nonhematopoietic cells and their effects have only recently been implicated in disease pathogenesis and are poorly characterized, but they are required for TSST-1 penetration of the vaginal epithelium and TSS development in rabbits (8, 17, 19, 20).

Endothelial cell responses critically contribute to early innate immune system activation. This is central to both immune protective and pathological responses that play a role in the pathogenesis of various cardiovascular diseases, such as atherosclerosis (21, 22), and possibly IE. In vascular endothelial cells, TSST-1 suppresses autophagy, indicating its ability to modify endothelial function for S. aureus intracellular survival or persistence (23). However, an important component of the toxicity associated with SAgs in both humans and rabbits is the resulting enhanced susceptibility to host-derived (endogenous) endotoxin (lipopolysaccharide [LPS]) (24). Endogenous LPS is the primary agent driving morbidity and mortality in experimental TSS (25, 26). While not completely defined, the TSST-1 and LPS synergistic activation of Kupffer cells is known to impair the liver's detoxification capabilities (27, 28) and activation of peripheral blood mononuclear cells and splenocytes enhances production of inflammatory cytokines (29, 30). Together, these events partially contribute to the clinical systemic effects of TSS. The physiologic impact of TSST-1 on the vascular endothelium in the presence and absence of LPS is largely unknown. Hence, we directly addressed whether TSST-1 alone or in combination with LPS impairs critical cellular functions as a mechanism for IE development.

We developed, characterized, and utilized immortalized human aortic endothelial cells (iHAECs) as an *in vitro* large-vessel model system. We provide evidence that TSST-1 interaction with iHAECs increases both protein expression of endothelial cell adhesion molecules and endothelial monolayer permeability for water-soluble molecules. In addition, the specific interaction of TSST-1 with iHAECs decreases reendothelialization and suppresses production of the chemokine interleukin-8 (IL-8). TSST-1 also inhibits IL-8 production in iHAECs stimulated with LPS, and this inhibition cannot be rescued with tumor necrosis factor alpha (TNF-α) or IL-1β pretreatment. The TSST-1 suppressive phenotype is not dependent on the MHC-II-binding domain of TSST-1. The differential expression of cell-bound and secreted markers of vascular endothelial cell activation may play an important role in promoting vegetation growth while dampening immune responses to S. aureus within vegetations and adjacent vascular tissues.

## RESULTS

### iHAECs phenotypically and functionally closely resemble pHAECs.

**FIG 1:**
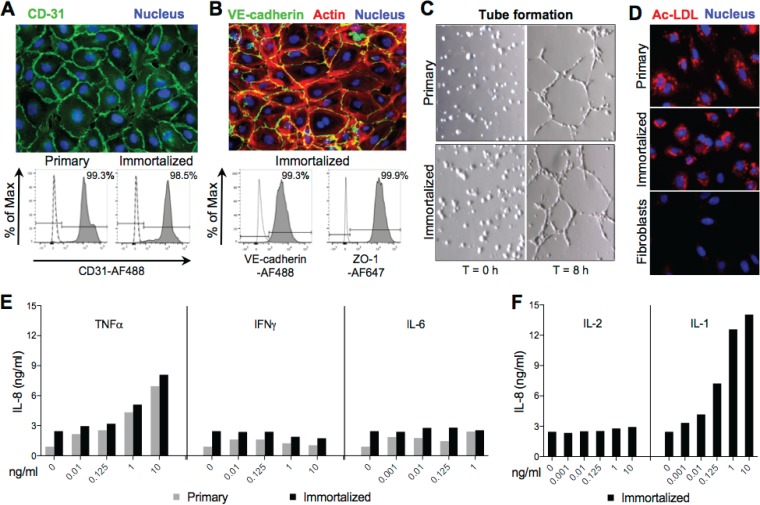
Immortalized HAECs retain phenotypic and functional characteristics of primary cells. (A) (Top) Immortalized HAEC confluent monolayers stained with CD31-AF488 antibody (green) and DAPI (blue). (Bottom) Flow cytometry histogram plots of primary or immortalized HAECs labeled with CD31-AF488 antibody. Secondary antibody only (dashed line) and nonstained cells (dotted line) were used as negative controls. (B) (Top) Immortalized HAEC confluent monolayers stained with VE-cadherin-AF488 (green) antibody, Acti-Stain 555 phalloidin (red), and DAPI (blue). (Bottom) Flow cytometry histogram plots of immortalized HAECs labeled with VE-cadherin-AF488 and ZO-1-AF647 antibodies. Secondary antibody only (dashed line) was used as a negative control. (C) Tube forming assay of primary (top) and immortalized (bottom) HAECs at 0 h and 8 h after seeding in Matrigel-coated wells. (D) Acetylated low-density lipoprotein (Ac-LDL) uptake assay of primary HAECs (top), immortalized HAECs (middle), and fibroblasts (negative control; bottom) treated with fluorescently labeled Ac-LDL (red) for 2.5 h and stained with DAPI (blue). (E) IL-8 detection in culture supernatants 24 h after treatment of primary or immortalized HAECs with increasing concentrations of TNF-α, IFN-γ, or IL-6. (F) IL-8 detection in culture supernatants 24 h after treatment of immortalized HAECs with increasing concentrations of IL-2 or IL-1.

It is challenging to address mechanistic questions in primary cell cultures, owing to their short life span, batch-to-batch variability, and high cost. To overcome these limitations, we developed iHAECs and thoroughly characterized the cell line both phenotypically and functionally. Confluent monolayers of both primary HAECs (pHAECs) and iHAECs exhibited a cobblestone morphology characteristic of vascular endothelial cells (Fig. 1A and B, top images) and expressed similar levels of CD-31/PECAM-1 (an intercellular adhesion molecule expressed on the surface of endothelial cells) (31), assessed by flow cytometry (Fig. 1A, bottom image). iHAECs expressed high levels of vascular endothelial (VE) cadherin and zonula occludens protein 1 (ZO-1), components of intercellular adherens and tight junctions, respectively, critical for maintaining endothelial barrier function (Fig. 1B). The cells also expressed high levels of endothelial nitric oxide synthase (eNOS), critical for endothelial cell homeostasis, and high levels of von Willebrand factor (vWF) (see Fig. S1A in the supplemental material), a commonly used endothelial cell marker expressed almost exclusively on endothelial cells (31). Organization of actin fibers in iHAEC monolayers was consistent with those of large artery endothelial cells (32), where F-actin fibers localized centrally and longitudinally with peripherally localized F-actin bundles (Fig. 1B, top image). We also performed flow cytometry analyses of iHAEC expression of selected endothelial cell receptors and coreceptors critical for activation and amplification of immune responses. Approximately 95% of cells expressed low to moderate levels of MHC-II and CD40, and all cells expressed high levels of Toll-like receptor 4 (TLR4) and CD14, LPS receptor and coreceptor, respectively (Fig. S1B).

We then tested whether immortalized cells were functionally similar to primary cells. Both pHAECs and iHAECs formed capillary-like structures (tubes) in Matrigel-coated wells (Fig. 1C), thus modeling the reorganization stage of angiogenesis (proliferation, migration, and tube formation) critical for vascular remodeling. Uptake of low-density lipoproteins (LDL) is another characteristic of endothelial cells that has routinely been used to isolate endothelial cells from mixed cultures (33). To test for LDL uptake, iHAECs were treated with fluorescently labeled acetylated LDL (Ac-LDL) in culture for 2.5 h and imaged by fluorescence microscopy. pHAECs and iHAECs exhibited comparable phagocytic activities, observed as a fluorescent Ac-LDL punctate pattern with perinuclear distribution (Fig. 1D). No Ac-LDL endocytosis was observed in fibroblasts, used as negative control. Endothelial cells are known to respond to inflammatory stimuli, such as the cytokines TNF-α and IL-1β, by producing proinflammatory molecules such as the chemokine IL-8 (34). Thus, we tested IL-8 production in pHAECs (Fig. 1E) and iHAECs (Fig. 1E and F) in response to increasing concentrations of proinflammatory cytokines. Consistent with the known physiology of endothelial cells, pHAECs and iHAECs treated with TNF-α and IL-1β produced IL-8 in a dose-dependent manner while remaining unresponsive to gamma interferon (IFN-γ), IL-6, and IL-2 stimulation (Fig. 1E and F). Together, these results indicate that iHAECs retain phenotypic and functional characteristics of primary large-vessel endothelial cells and hence are a useful model system in which to address questions relevant to vascular endothelial function.

### TSST-1 induces activation of iHAECs.

**FIG 2:**
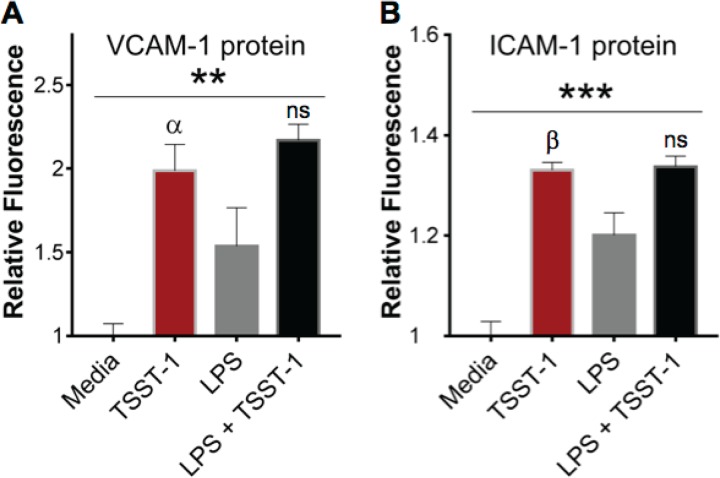
TSST-1 directly activates iHAECs. Shown is In-Cell Western assay detection of total protein of the cell adhesion molecules VCAM-1 (A) and ICAM-1 (B) in iHAECs treated with TSST-1 (25 μg/ml) or LPS (1 to 2 ng/ml), individually and in combination for 24 h. *P* values were determined by one-way ANOVA (asterisks) with Holm-Sidak's multiple-comparison test shown for TSST-1 versus medium or versus LPS plus TSST-1 (Greek letters, adjusted *P* value). (A) **, *P* = 0.003; α, *P* = 0.005 (TSST-1 versus medium). (B) ***, *P* = 0.0001; β, *P* = 0.0003 (TSST-1 versus medium). (A and B) TSST-1 versus LPS plus TSST-1 is not significant (ns).

For the studies described herein, we strived to use physiologically relevant concentrations of toxins. TSST-1-encoding S. aureus strains produce between 5 and 100 μg/ml of TSST-1 in liquid culture and up to 16,000 μg/ml in biofilms (35). Vegetations on aortic valves are, in fact, tissue biofilms that support bacterial growth as high as 5 × 10^9^ CFU in experimental IE (8, 9). Based on these data, we expect endothelial cells in close proximity of vegetative lesions to be exposed to relatively high levels of toxins produced by growing bacteria. Hence, unless specified otherwise, we used TSST-1 at concentrations between 12 and 25 μg/ml. LPS was used at concentrations between 0.5 and 2.5 ng/ml. These LPS concentrations were previously measured in the bloodstream of patients with liver disease and pneumonia, yet they are lower than those in patients with S. aureus TSS (4 ng/ml) (25, 36–38). iHAECs were stimulated with TSST-1, LPS, or LPS plus TSST-1 for 24 h and examined for markers of cellular activation. Endothelial cell activation is defined by the upregulation of cell surface adhesion molecules important for recruitment and transmigration of circulating leukocytes (39), specifically vascular cell adhesion molecule 1 (VCAM-1) and intercellular adhesion molecule 1 (ICAM-1). TSST-1 and LPS individually induced a significant increase in total cellular levels of VCAM-1 and ICAM-1 measured by the In-Cell Western assay, while no significant change above that of TSST-1 alone was measured with simultaneous stimulation with LPS and TSST-1 (Fig. 2). These results provide evidence that TSST-1 directly activates vascular endothelial cells.

### TSST-1 increases permeability of the iHAEC monolayer.

Proinflammatory stimuli activate endothelial cells and disrupt the endothelial barrier, a process that promotes vascular inflammation and extravasation of fluids (6, 40). Since TSST-1 activates iHAECs, we next examined whether TSST-1 induces changes in endothelial monolayer permeability. For this, confluent iHAEC monolayers grown on Transwell inserts were stimulated with TSST-1, LPS, or LPS plus TSST-1 for 24 h and examined for leakage of fluorescent tracers with low or high molecular weight (Lucifer yellow [LY] and bovine serum albumin [BSA], respectively) through the monolayer. LY moves across the endothelial cell barrier primarily in a paracellular manner (via gaps in the monolayer's tight junctions). BSA, due to its larger size, moves across the healthy endothelium primarily by the active transcellular pathway mediated by caveolae and transcytosis and, in addition, via the paracellular pathway upon disturbances to tight, gap, and/or adherens junctions (41). EDTA chelates extracellular calcium causing leakage of the endothelial barrier and thus is commonly used as a positive control.

**FIG 3:**
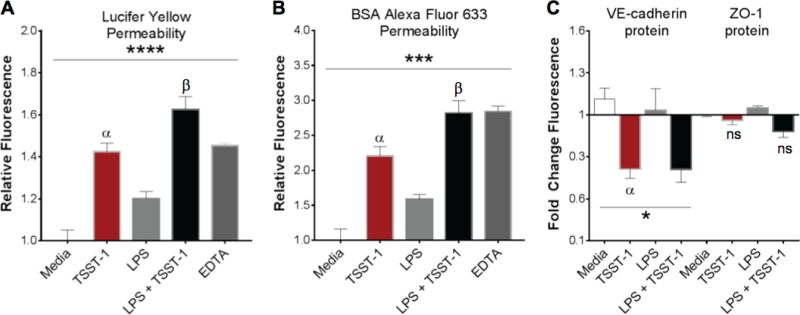
TSST-1 disrupts iHAEC monolayer barrier function. (A and B) Leakage of fluorescent tracers across a confluent iHAEC monolayer stimulated for 24 h with TSST-1 (25 μg/ml) or LPS (1 ng/ml), individually and in combination: Lucifer yellow (low-molecular-weight tracer) (A) and BSA-Alexa Fluor 633 (high-molecular-weight tracer) (B). EDTA (50 mM) was used as a positive control. (C) In-Cell Western assay total protein detection of VE-cadherin and ZO-1 in confluent iHAEC monolayers stimulated for 24 h with TSST-1 (25 μg/ml) or LPS (1 ng/ml), individually and in combination. *P* values were determined by one-way ANOVA (asterisks) with Holm-Sidak's multiple-comparison test (Greek letters, adjusted *P* value). (A) ****, *P* < 0.0001; α, *P* = 0.001 (TSST-1 versus medium); β, *P* = 0.04 (TSST-1 versus LPS plus TSST-1). (B) ***, *P* = 0.0001; α, *P* = 0.001 (TSST-1 versus medium); β, *P* = 0.05 (TSST-1 versus LPS plus TSST-1). (C) VE-cadherin: *, *P* = 0.01; α, *P* = 0.05 (TSST-1 versus medium). ZO-1: ns, not significant (TSST-1 versus medium or versus LPS plus TSST-1).

We observed that TSST-1 and LPS individually induced leakage of both low- and high-molecular-weight tracers across the iHAEC monolayer (Fig. 3A and B). Simultaneous stimulation with LPS and TSST-1 resulted in permeability changes consistent with an additive effect. Because vascular endothelial permeability is regulated primarily by the adherens junction protein VE-cadherin with involvement of ZO-1, a scaffold protein of endothelial tight junctions (41), we measured protein expression of VE-cadherin and ZO-1 in toxin-stimulated cells. TSST-1 stimulation significantly decreased VE-cadherin protein expression, while expression of ZO-1 was not significantly changed due to TSST-1 stimulation (Fig. 3C). Under our experimental conditions, the decreased VE-cadherin protein expression in costimulated cells was driven primarily by TSST-1 (Fig. 3C). While the In-Cell Western assay is a powerful tool that provides protein expression levels on cell populations with a spatial resolution down to 21 μm (the estimated size of HAECs is between 20 to 50 μm), it lacks single-cell resolution. Therefore, we examined VE-cadherin distribution in iHAEC monolayers by fluorescence microscopy. iHAEC treatment with TSST-1 results in discontinuous distribution of VE-cadherin, suggesting a loss of tight adherens junctions on the cell-cell interface. In addition, TSST-1 induced an overall decrease in VE-cadherin fluorescence intensity by microscopy (Fig. S2), consistent with a decrease in VE-cadherin protein levels by the In-Cell Western assay (Fig. 3C). Together, the data suggest that TSST-1 alters iHAEC monolayer barrier function by a mechanism dependent on VE-cadherin redistribution, degradation, or decreased production.

### TSST-1 impedes iHAEC monolayer wound closure.

**FIG 4:**
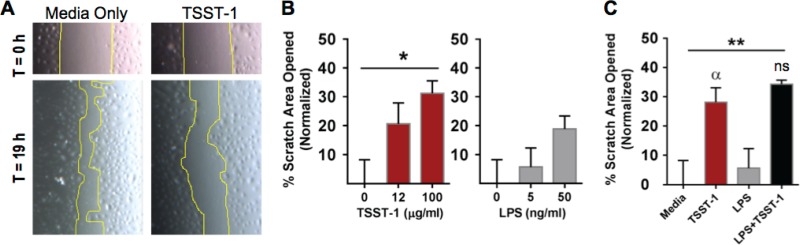
TSST-1 impedes iHAEC monolayer wound closure. Shown are results of an *in vitro* wound healing assay with immortalized HAECs stimulated with TSST-1 and/or LPS for 19 h. (A) Representative phase-contrast images of scratched iHAEC monolayers with or without TSST-1 (12.5 μg/ml) at 0 h (top) and 19 h (bottom). (B) Quantification of the scratched area in monolayers treated with increasing concentrations of TSST-1 or LPS. *P* values were determined by one-way ANOVA. *, *P* = 0.04. (C) Quantification of the scratched area in monolayers treated with TSST-1 (12.5 μg/ml) or LPS (5 ng/ml), individually and in combination. *P* values were determined by one-way ANOVA (asterisks) with Holm-Sidak's multiple-comparison test (Greek letters, adjusted *P* value). **, *P* = 0.009; α, *P* = 0.04 (TSST-1 versus medium); ns, not significant (TSST-1 versus LPS plus TSST-1).

Reendothelialization is essential for vascular endothelial repair (40). To examine the effects of TSST-1 on endothelialization, we utilized an *in vitro* wound healing assay. For this, mechanical damage was introduced by means of a scratch in confluent iHAEC monolayers before stimulation with various concentrations of TSST-1 or LPS, or monolayers were left untreated for up to 19 h (Fig. 4A and B). During this time, cells at the wound edges stimulated by loss of monolayer integrity proliferate and migrate to close the gap. At 19 h, the area of the scratch remaining open was measured and normalized to the scratch area at 0 h. TSST-1 and LPS alone significantly inhibited reendothelialization, with up to 28% and 6% of the scratched areas remaining open, respectively (Fig. 4C). In the scratched monolayers treated with toxins simultaneously (TSST-1 at 12 μg/ml and LPS at 5 ng/ml), the overall delay in monolayer wound healing was mediated primarily by TSST-1 (Fig. 4C). These results provide evidence that TSST-1 disrupts reendothelialization and suggest that vascular exposure to TSST-1 interferes with a crucial vascular endothelial function—remodeling and repair.

### TSST-1 inhibits IL-8 production in iHAECs.

**FIG 5:**
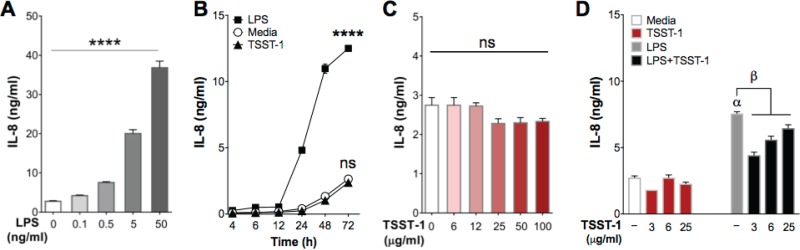
TSST-1 stimulation of iHAECs does not induce IL-8 secretion. (A) iHAECs stimulated with increasing concentrations of LPS (0.1 to 50 ng/ml) for 24 h. *P* value was determined by one-way ANOVA. ****, *P* < 0.0001. (B) iHAECs stimulated with TSST-1 (25 μg/ml) or LPS (2.5 ng/ml) for 4 to 72 h. One-way ANOVA linear trend, *P* < 0.0001. (C) iHAECs stimulated with increasing concentrations of TSST-1 (6 to 100 μg/ml) for 24 h. (D) iHAECs stimulated for 24 h with TSST-1 (3, 6, or 25 μg/ml) alone and in the presence of LPS (0.5 ng/ml). *P* values were determined by one-way ANOVA with Holm-Sidak's multiple-comparison test (Greek letters, adjusted *P* value). α, *P* < 0.0001 (LPS versus medium); β, *P* < 0.01 (LPS versus LPS plus TSST-1).

Upon stimulation, the activated endothelium produces proinflammatory cytokines and chemokines to amplify immune responses, thus contributing to local or systemic inflammation (34). To address the role of TSST-1 in inducing chemokine/cytokine secretion in endothelial cells, we performed dose-response and time course stimulation experiments and measured IL-8 secreted in culture supernatants by enzyme-linked immunosorbent assay (ELISA). For dose-response analysis, cells were treated with increasing concentrations of TSST-1 (6 to 100 μg/ml) or LPS (0.1 to 50 ng/ml) for 24 h. For time course analysis, cells were treated with LPS (2.5 ng/ml) or TSST-1 (25 μg/ml) for 4 to 72 h. Consistent with published data, LPS stimulation induced IL-8 production in a dose- and time-dependent manner (Fig. 5A and B). In contrast, TSST-1 failed to induce IL-8 even at doses as high as 100 μg/ml (Fig. 5C) and during stimulation lasting as long as 72 h (Fig. 5B). Furthermore, iHAECs stimulated simultaneously with TSST-1 (3, 6, or 25 μg/ml) and LPS (0.5 ng/ml) secreted significantly less IL-8 than those stimulated with LPS alone (Fig. 5D). Importantly, we confirmed that the absence or reduced levels of IL-8 in culture supernatants were not due to loss of iHAEC viability upon stimulation with TSST-1 or TSST-1 plus LPS (Fig. S3A and B).

**FIG 6:**
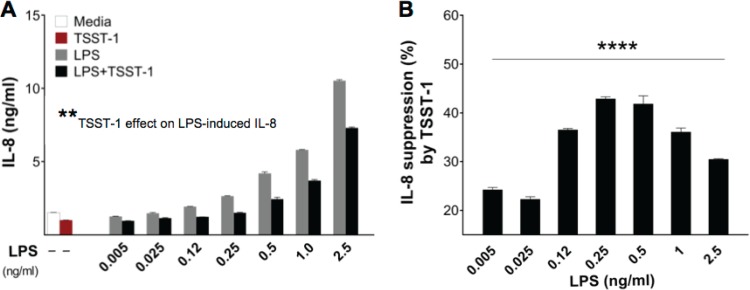
TSST-1 suppresses IL-8 responses to LPS in iHAECs. (A) iHAECs stimulated for 24 h with increasing concentrations of LPS (0.005 to 2.5 ng/ml) in the presence of TSST-1 (3 μg/ml). *P* values were determined by two-way ANOVA (asterisks). **, *P* = 0.002 (LPS versus LPS plus TSST-1 across all concentrations). (B) Percent suppression of LPS-mediated IL-8 secretion resulting from concomitant stimulation with TSST-1. *P* values were determined by one-way ANOVA. ****, *P* = 0.002.

Given these results, we asked whether TSST-1 iHAEC stimulation inhibits IL-8 production or fails to induce it. To directly answer this question, iHAECs were stimulated with increasing concentrations of LPS (0.005 to 2.5 ng/ml) in the presence of TSST-1 (3 μg/ml). TSST-1 consistently and significantly inhibited LPS-induced IL-8 production (Fig. 6A). When the TSST-1-mediated inhibition was expressed as percent suppression, the data exhibited a bell-shaped curve (Fig. 6B), suggesting that there is an LPS/TSST-1 ratio where TSST-1 exerts the most potent IL-8 inhibitory effect. Importantly, the TSST-1 inhibitory effect on LPS-induced IL-8 was confirmed in pHAECs and was also observed with IL-6 production (Fig. S4). To examine whether prestimulation with either toxin is required to overcome TSST-1-mediated inhibition, we prestimulated iHAECs with LPS or TSST-1 for 4 h prior to stimulation with the other toxin. We found that TSST-1 prestimulation recapitulated the IL-8 inhibitory effect of simultaneously adding TSST-1 and LPS. However, prestimulation with LPS significantly abrogated the inhibitory effect (Fig. S5). Together, the data suggest that the signaling pathways activated in iHAECs upon TSST-1 stimulation interfere with LPS signaling.

### TSST-1 IL-8 suppression is independent of MHC-II binding.

**FIG 7:**
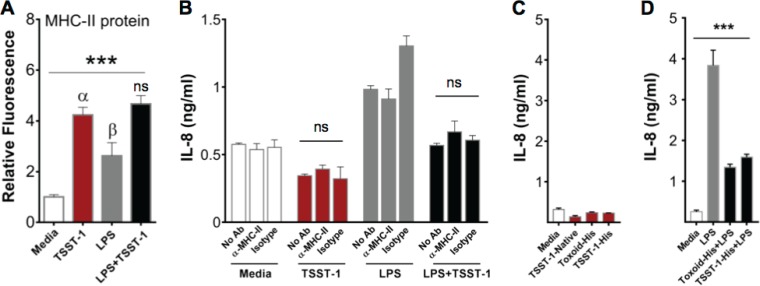
TSST-1 IL-8 suppression is independent of MHC-II binding. (A) In-Cell Western assay detection of MHC-II in iHAECs treated with TSST-1 (25 μg/ml) or LPS (1 ng/ml), individually and in combination for 24 h. Data are reported as fluorescence intensity normalized to that of medium-treated cells. *P* values were determined by one-way ANOVA (asterisks) with Holm-Sidak's multiple-comparison test (Greek letters, adjusted *P* value). ***, *P* < 0.0003; α, *P* = 0.0009 (TSST-1 versus medium); β, *P* = 0.03 (LPS versus medium); ns, not significant (TSST-1 versus LPS plus TSST-1). (B) iHAECs treated with an MHC-II-blocking antibody or isotype control antibody (10 μg/ml) for 30 min prior to stimulation with TSST-1 (10 μg/ml) and/or LPS (0.5 ng/ml) for 24 h also in the presence or absence of antibodies. Significance was determined by one-way ANOVA. (C) iHAECs stimulated for 24 h with His tag-purified toxoid (toxoid-His; MHC-II and TCR binding site inactivated), control His tag-purified TSST-1 (TSST-1–His), and native TSST-1 at 25 μg/ml each. (D) iHAECs stimulated with toxoid-His or TSST-1–His (2.4 μg/ml each) in the presence of LPS or LPS alone (1 ng/ml). Statistical analysis was done by one-way ANOVA with Holm-Sidak's multiple-comparison test. ***, *P* < 0.0001.

TSST-1 suppression of IL-8 in iHAECs stands in stark contrast to results obtained with immortalized human vaginal epithelial cells (iHVECs), in which TSST-1 induces expression of IL-8 (15, 16). Vaginal epithelial and vascular endothelial cells differ in physiological location and function, and this may dictate the differential responses when exposed to the same stimuli. In addition, iHAECs and iHVECs differ greatly in expression of the TSST-1 receptor MHC-II, where <2% of iHVECs are MHC-II positive (15, 19), while about 95% of iHAECs express MHC-II. Furthermore, TSST-1 stimulation of iHAECs induced about a 3-fold increase in MHC-II protein expression (Fig. 7A), while TSST-1 iHVEC stimulation had no effect on MHC-II expression (15). Therefore, we asked whether TSST-1 binding to MHC-II may mediate IL-8 suppression in iHAECs. To address this question, we incubated iHAECs with an MHC-II-blocking antibody prior to stimulation with TSST-1, LPS, or LPS plus TSST-1 and measured the IL-8 concentrations in supernatants (14). An isotype-matched antibody was used as a control. MHC-II blocking did not promote IL-8 production in TSST-1-stimulated cells, nor did it affect TSST-1-mediated suppression in cells stimulated with LPS plus TSST-1 (Fig. 7B). To confirm these results, we stimulated iHAECs with a well-characterized TSST-1 toxoid (G31S/S32P/H135A) inactive in MHC-II/TCR binding (42–44). Because TSST-1 toxoid was His tagged at the C terminus for purification purposes (toxoid-His), TSST-1 was similarly His tagged and purified (TSST-1–His), and both native TSST-1 (used throughout our studies) and TSST-1-His were used as controls. Native TSST-1, TSST-1–His, and toxoid-His failed to induce IL-8 secretion in iHAECs (Fig. 7C), even at doses as high as 200 μg/ml (Fig. S3C). Furthermore, toxoid-His also suppressed IL-8 in LPS-costimulated iHAECs in a manner similar to that of native and His-tagged TSST-1 (Fig. 7D), providing evidence that TSST-1 inhibits IL-8 secretion upon LPS stimulation via a mechanism independent of MHC-II binding.

### TNF-α and IL-1β pretreatment does not rescue iHAECs from TSST-1-mediated IL-8 suppression.

**FIG 8:**
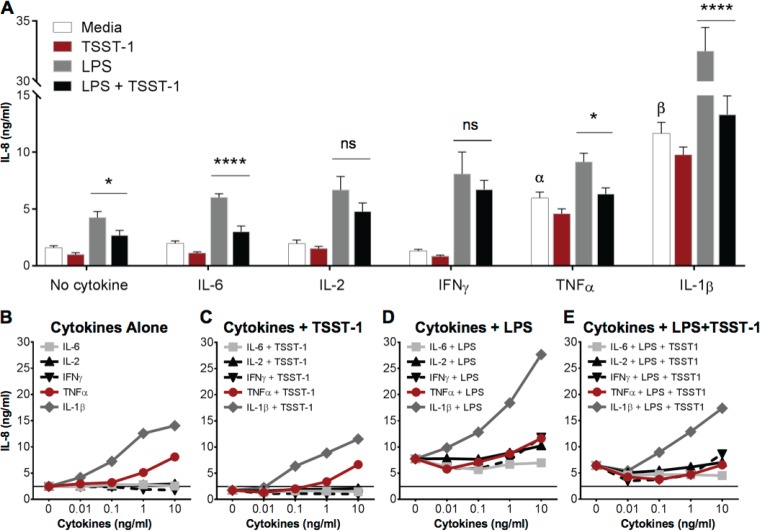
TNF-α and IL-1β pretreatment does not rescue iHAECs from TSST-1-mediated IL-8 suppression during simultaneous LPS stimulation. (A) iHAECs treated for 24 h with IL-2, IFN-γ, TNF-α, or IL-1β (10 ng/ml) or IL-6 (1 ng/ml) before stimulation with TSST-1 (12.5 μg/ml) or LPS (0.5 ng/ml), individually and in combination for an additional 24 h. (B to E) iHAECs treated for 24 h with increasing concentrations of IL-2, IFN-γ, TNF-α, or IL-1β (0.01 to 10 ng/ml) or IL-6 before stimulation with TSST-1 (12.5 μg/ml) or LPS (0.5 ng/ml), individually and in combination for an additional 24 h (all concentrations of IL-6 are 1 order of magnitude lower than those of other cytokines: “0.01” on the *x* axis = 0.001 ng/ml of IL-6; “10” on the *x* axis = 1 ng/ml of IL-6). iHAECs' baseline production of IL-8 under all conditions (2 ng/ml) is indicated by a horizontal line. (B) Cytokine treatment alone (medium-only control). (C) Cytokine-pretreated iHAECs stimulated with TSST-1. (D) Cytokine-pretreated iHAECs stimulated with LPS. (E) Cytokine-pretreated iHAECs stimulated with LPS plus TSST-1. *P* values were determined by one-way ANOVA with Holm-Sidak's multiple-comparison test (asterisks, adjusted *P* value for LPS versus LPS plus TSST-1; Greek letters, adjusted *P* value for cytokine only versus medium alone when significant). No cytokine, *, *P* = 0.03; IL-6, ****, *P* < 0.0001; IL-2 and IFN-γ, not significant (ns); TNF-α, *, *P* = 0.01; IL-1β, ****, *P* < 0.0001. α (TNF-α), *P* = 0.05, β (IL-1β), *P* < 0.0001.

Cytokine pretreatment was reported to be required for effective endothelial cell activation and IL-8 secretion (14, 45, 46). Therefore, we examined the effect of pretreatment with IL-6, IL-2, IFN-γ, TNF-α, or IL-1β on IL-8 production after subsequent TSST-1 stimulation alone or in combination with LPS (Fig. 8A). iHAECs were pretreated with cytokines for 24 h prior to TSST-1 or LPS stimulation. Consistent with previous reports, TNF-α and IL-1β significantly increased IL-8 secretion in LPS-stimulated cells. Yet none of the cytokine pretreatments induced iHAECs to secrete IL-8 upon TSST-1 stimulation above the level of cytokine treatment alone (Fig. 8B and C). Cytokine pretreatment followed by LPS plus TSST-1 resulted in differential cytokine-specific IL-8 responses. Pretreatment with IFN-γ or IL-2 significantly relieved TSST-1-mediated suppression of IL-8 after LPS costimulation. In contrast, pretreatment with IL-6, TNF-α, or IL-1β did not abrogate the suppressive effect of TSST-1 on LPS-induced IL-8. TSST-1 interference with LPS signaling leading to IL-8 production dominated the responses despite iHAEC sensitivity to the activating effects of TNF-α or IL-1β. We tested the effects of cytokine pretreatment on IL-8 production across a concentration range of up to 10 ng/ml (1 ng/ml for IL-6) and consistently observed a dose-dependent increase in IL-8 secretion under TNF-α or IL-1β with or without LPS stimulation (Fig. 8B to E). iHAECs responded to IL-1β and TNF-α at concentrations above 0.01 ng/ml and 0.1 ng/ml, respectively (Fig. 8B); addition of TSST-1 to cytokine-pretreated cells did not significantly affect IL-8 secretion (Fig. 8C). iHAECs were sensitive to IL-1β prestimulation across various concentrations, enhancing LPS-mediated IL-8 production by ∼10-fold at the highest dose (10 ng/ml). IFN-γ significantly enhanced IL-8 production only at 10 ng/ml (Fig. 8D). Taken together, significant TSST-1 inhibition of IL-8 production with LPS costimulation occurred across tested concentrations in iHAECs pretreated with IL-6, TNF-α, or IL-1β, with IL-2- and IFN-γ-pretreated cells exhibiting IL-8 production not significantly different than LPS treatment alone (Fig. 8E). These data confirm that TSST-1 exerts a suppressive effect on IL-8 production despite prestimulation with potent proinflammatory stimuli such as TNF-α and IL-1β.

## DISCUSSION

Vascular endothelial cells perform diverse functions essential for physiological cardiovascular processes and vascular homeostasis; thus, they are highly dynamic and plastic in their responsiveness to environmental signals (6, 34). Endothelial cells lining the vasculature are exposed to a great variety of blood-borne stimuli and are in fact one of the first cell types to detect and respond to these stimuli, including circulating pathogens and their toxins (1, 6, 13, 47). Endothelial cell responses critically contribute to early innate immune system activation. This is central to both immune protective and pathological responses that may play a role in the pathogenesis of various cardiovascular diseases (12, 14, 34). S. aureus IE is an aggressive and fast-progressing infection of the cardiac endothelium (1–3). Current evidence indicates that the SAgs SEC, TSST-1, and the *egc* toxins play a novel and essential role in the etiology of IE caused by S. aureus (8, 9). Studies of experimental IE suggest that SAgs exert their effects at the infection site to cause tissue pathology and promote vegetation growth by yet-unidentified mechanisms (9). In this study, with the use of iHAECs, we obtained evidence that TSST-1 exerts its effects directly on vascular endothelial cells, resulting in an activated phenotype, increased monolayer permeability, and delayed reendothelialization. These results highlight a key role for TSST-1 in initiating pathological changes in the endothelium that can be central to the pathogenesis of S. aureus IE.

Proinflammatory mediators such as thrombin and histamine disrupt the endothelial barrier by binding to G-protein-coupled receptors, activating and accumulating matrix metalloproteinases in intercellular junctions (41, 48). In epithelial cells, TSST-1 was shown to activate ADAM17 (a disintegrin and metalloproteinase 17) via interactions with a G-protein-coupled receptor (16). In endothelial cells, vascular integrity and permeability barrier function are largely regulated by VE-cadherin and associated α-, β-, and p120-catenin adhesion complexes, key structural components of adherens junctions (40, 41). In our studies, TSST-1 iHAEC stimulation decreased total cellular VE-cadherin, consistent with increased BSA leakage via the intercellular and paracellular pathway and indicating that the integrity of the VE-cadherin adhesion complex was compromised. VE-cadherin adherens junctions are also stabilized by dynamic microtubules in areas of cell-cell contact (40, 41). Thrombin, via heterotrimeric G-protein activation, depolymerizes microtubules at the cell periphery, initiating endothelial barrier dysfunction (49). Proatherogenic stimuli and oxidative stress also chemically modify α-tubulin (an essential component of microtubules), causing microtubule reorganization and increased vascular permeability (40, 41). It remains to be elucidated whether TSST-1 uses similar molecular mechanisms to control VE-cadherin turnover in endothelial cells.

Of interest, dynamic microtubules also control cell movement and migration. Hyperacetylation of α-tubulin increases microtubular stability, causing endothelial cell arrest or limited mobility (50). Vascular repair is dependent on endothelial cell migration (as well as proliferation). Interfering with any of these processes inhibits reendothelialization of a damage site. Endothelial injury, whether due to mechanical damage or toxicity caused by S. aureus colonization, can promote IE if the damaged site is prevented from healing (8). We show that iHAECs treated with TSST-1 were impaired in their ability to restore the integrity of the monolayer after mechanical disruption. Hence, it is possible that TSST-1 triggers both an increase in iHAEC monolayer permeability and a decrease in wound healing by interfering with α-tubulin dynamics. Future studies will be required to determine the exact mechanism of TSST-1 dysregulation of vascular permeability and repair. However, destabilization of the endothelium via barrier dysfunction and inhibition of reendothelialization exposes the subendothelial tissues, which can promote accumulation of fibrin, other procoagulation factors, and S. aureus colonization, which together contribute to vegetation growth (1, 51).

In stark contrast with results obtained with epithelial cells and adipocytes (15, 16, 18, 19), TSST-1 failed to induce IL-8 and IL-6 production in HAECs (both primary and immortalized) and suppressed production of these mediators from cells concurrently stimulated with LPS. iHAECs were refractive to the effects of TSST-1 even after pretreatment with proinflammatory cytokines such as TNF-α and IL-1β. Furthermore, TSST-1-mediated suppression of LPS-induced IL-8 persisted despite pretreatment with TNF-α and IL-1β. The TSST-1 receptor on nonhematopoietic cells is not known with certainty, and we provide evidence that MHC-II binding does not mediate the suppressive effects on IL-8 secretion. However, the inhibitory effect of TSST-1 on LPS signaling provides insight into cellular pathways uniquely activated by TSST-1. Recently, p120-catenin (VE-cadherin-interacting partner) was found to not only contribute to the stability of adherens junctions but also function as an endogenous negative regulator of LPS-mediated inflammation (52–54). Pools of p120-catenin exist also in the cytoplasm and the nucleus, where they exhibit pleiotropic functions associated with cell adhesion, actin dynamics, and cell signaling. Cytoplasmic p120-catenin blocks nuclear factor κB (NF-κB) activation by inhibiting the activity of the Rho-GTPase RhoA and preventing LPS/TLR-4 association with its adaptor myeloid differentiation primary response gene 88 (MyD88) (52–54).

Upon LPS stimulation, activated Rho-GTPases phosphorylate p120-catenin and other junction proteins, resulting in protein degradation and consequently increases in both endothelial permeability and NF-κB activation (IL-8 gene transcription) (55). However, if p120-catenin is overexpressed in endothelial cells (increasing the cytoplasmic pool), NF-κB activation is suppressed upon LPS stimulation (52, 54). Therefore, we propose that TSST-1 suppresses LPS-induced IL-8 via a pathway that includes an increased cytoplasmic pool of p120-catenin that, in turn, prevents derepression of RhoA. Consistent with this hypothesis, iHAECs pretreated with IFN-γ, known to induce p120-catenin phosphorylation, exhibit no significant IL-8 suppression with stimulation with LPS plus TSST-1 (the effect of IL-2 on p120-catenin phosphorylation has not been documented) (56). Furthermore, if iHAECs are pretreated with LPS, TSST-1 no longer suppresses IL-8 production, suggesting that once RhoA is activated, the TSST-1-mediated pathway can no longer exert its inhibitory effect. These scenarios are speculative, and studies to address these hypotheses are under way.

While TSST-1 does not induce IL-8 and IL-6 *in vitro*, it upregulates expression of VCAM-1 and ICAM-1, cell surface molecules that mediate adhesion and transmigration of leukocytes across the endothelium (1, 6, 39). In contrast to our results in iHAECs, HUVEC stimulation with TSST-1 for 4 to 6 h was not sufficient to induce ICAM-1 and VCAM-1 expression (12). These studies used SAgs in the nanogram-per-milliliter range, concentrations that are sufficient to induce T cell proliferation but likely suboptimal for inducing responses in vascular endothelial cells, which may be exposed to higher concentrations of SAgs produced locally at an infection site *in vivo*. Endothelial cells originating from different vascular beds are also widely heterogeneous in phenotype, function, and sensitivity to stimuli (34, 57). Therefore, the strength of our model lies within the origin of our cell line, aortic endothelium important in S. aureus IE pathophysiology. Expression of VCAM-1 and ICAM-1 is a hallmark of the activated endothelium (34, 39). Importantly, we documented that in iHAECs TSST-1 differentially regulates expression of activation markers associated with leukocyte trafficking (VCAM-1 and ICAM-1) and those associated with leukocyte chemoattraction and activation (IL-8 and IL-6). These apparent contradictive effects may serve as a control mechanism to prevent an overt inflammatory response to various bacterial toxins *in vivo*, but they may also contribute to immune dysregulation during S. aureus infection and prevent elimination of bacterial pathogens from an infection site. In support of this, vegetative lesions from IE in rabbits show vascular endothelial and subendothelial tissues in near proximity to vegetations to be mostly devoid of neutrophils, which stands in stark contrast with large and numerous S. aureus clusters within vegetations (8).

In conclusion, S. aureus is known for causing IE in individuals without preexisting valvular disease, utilizing subclinical inflammatory or mechanical lesions in the endothelium for pathogen adhesion and colonization (1, 2). In SAg-mediated responses, immune cell priming (superantigenicity) is placed as a triggering event mediating subsequent endothelial cell activation (11, 13). We developed and characterized iHAECs as a large-vessel model system to examine the effects of SAgs directly on vascular endothelial function. The newly developed iHAECs retain phenotypic and functional characteristics of primary cells. The results presented herein highlight the role of SAgs in the alteration of vascular endothelial cell phenotype and function independent of TSST-1 superantigenic activity and in the absence of proinflammatory cytokines. Endothelial dysfunction is a pathological condition with multiple manifestations, including increased expression of cellular adhesion molecules and/or cytokines/chemokines (endothelial cell activation), increased endothelial permeability (impaired vascular barrier function), or reduced endothelial cell proliferation and migration (impaired vascular healing and remodeling) (6, 34, 39). We provide evidence that TSST-1 induces vascular endothelial dysfunction. TSST-1 exploits the phenotypic and functional plasticity of the endothelium, where it activates cells to upregulate expression of adhesion molecules, downregulates and dysregulates expression of adherens junction proteins and IL-8/IL-6 production, and disrupts barrier function and reendothelialization, critical physiological properties of the endothelium. The iHAEC phenotypic and functional changes resulting from the action of SAgs can therefore directly contribute to the aggressive nature of S. aureus IE and development of life-threatening complications.

## MATERIALS AND METHODS

### Generation of iHAECs.

Cryopreserved primary human aortic endothelial cells (pHAECs) isolated from a deceased organ donor were recovered from liquid nitrogen and expanded in medium 200 supplemented with low-serum growth supplement (LSGS; Gibco, Life Technologies Corporation, Grand Island, NY) in 1% gelatin-coated 60-mm tissue culture plates (CELLTREAT Scientific Products, Pepperell, MA). Primary cells at the second passage were transduced with a combination of pBABE-Hygro-hTERT and pLXSN-HPV16 E6/E7 retroviruses overnight in 8 μg/ml of Polybrene, and one plate was mock transduced with Polybrene alone (58, 59). Subsequently, the cells were subcultured into 1% gelatin-coated 100-mm tissue culture plates and grown under hygromycin selection (12 μg/ml) for 10 days. Several clones isolated by ring cloning were subcultured for the next 12 days into a selection medium containing 100 μg/ml of neomycin. CD31 expression in several clones examined by flow cytometry showed that more than 98% of cells within each clone expressed high levels of the marker. Of the examined clones, a randomly selected immortalized HAEC (iHAEC) clone was expanded into an iHAEC line, and aliquots were cryopreserved in liquid nitrogen for repeated expansion and use. The selected clone was repeatedly subcultured at 80 to 90% confluence in medium 200 with LSGS that was changed every 2 to 3 days up to passage 80 to document the immortal properties of the cell line. All experiments described here were performed utilizing aliquots of a single clone at passages between 2 and 10. Cells were collected at near confluence by incubation with trypsin-EDTA (TE) that was neutralized with trypsin neutralizer (TN; both by Invitrogen, Life Technologies Corporation, Carlsbad, CA), washed in complete medium, counted by trypan blue exclusion, and utilized in downstream experiments.

### Flow cytometry.

The pHAECs or iHAECs grown in 1% gelatin-coated tissue culture plates up to about 90% confluence were collected with TE/TN. Cells were washed, fixed, permeabilized using the Fix/Perm kit (Invitrogen Corporation, Frederick, MD), and incubated with FcBlock (BD Biosciences, San Jose, CA) and 10% goat serum to block nonspecific staining. Cells were stained with the primary antibodies for 2 h on ice, washed, and incubated with the secondary fluorescently labeled antibodies for 2 h on ice, followed by flow cytometry analysis (BD LSR II; Becton Dickinson, Franklin Lakes, NJ). Data were analyzed with FlowJo (FlowJo, LLC, Ashland, OR). The following antibodies were used: mouse anti-human CD31 (clone JC/70A; Pierce/Thermo Fisher Scientific, Rockford, IL); CD40 (clone 5C3; BD Biosciences); MHC-II (clone TÜ39; BD Biosciences); VE-cadherin (clone 123413; R&D Systems, Minneapolis, MN); rabbit anti-human ICAM-1 (polyclonal antibody; NeoScientific, Woburn, MA); CD14, eNOS, TLR4, vWF, and ZO-1 (all polyclonal antibodies; Bioss Antibodies, Inc., Woburn, MA); and secondary antibodies goat anti-mouse Alexa Fluor 488 (AF488) and goat anti-rabbit Alexa Fluor 647 (AF647) (both from Molecular Probes, Eugene, OR).

### Fluorescence microscopy.

For CD31 and VE-cadherin immunofluorescence, iHAECs were grown to confluence on 1% gelatin-coated coverslips, fixed with cold methanol, washed, permeabilized with 0.1% Tween 20, blocked with 1% BSA and 10% animal species-specific serum of the secondary antibody, and stained with primary antibodies (CD31, clone JC/70A [Pierce/Thermo Fisher Scientific]; VE-cadherin, clone 123413 [R&D Systems]) and secondary fluorescent label-conjugated antibodies (goat anti-mouse AF488 or goat anti-rabbit AF647; Molecular Probes), with extensive washing between staining steps. Actin fibers were stained with Acti-Stain 555 phalloidin (Cytoskeleton, Inc., Denver, CO) (32). To demonstrate phagocytosis of acetylated low-density lipoprotein-DiI (Ac-LDL-DiI), pHAECs and iHAECs were grown on 1% gelatin-coated coverslips up to about 80% confluence, incubated in medium 200 with 0.5% BSA for 1 h at 37°C, then incubated with Ac-LDL-DiI for 2.5 h at 37°C, then incubated with medium 200-BSA for 20 min at 37°C, washed extensively between the steps, and finally fixed with phosphate-buffered formalin. Cells were mounted with ProLong gold antifade with 4′,6-diamidino-2-phenylindole (DAPI; Molecular Probes, Life Technologies, Carlsbad, CA) and imaged on an Olympus BX61 upright epifluorescence microscope equipped with cellSens software (Olympus Corporation). Images from different channels were pseudocolored and overlaid with ImageJ (NIH, Bethesda, MD).

### Tube forming assay.

pHAECs and iHAECs were plated into a Matrigel-coated (Corning Life Sciences, Tewksbury, MA) 24-well plate (40,000 cells/well) and incubated at 37°C and 5% CO_2_, and images were captured at several time points on a Nikon SMZ1500 stereoscope (Nikon Metrology, Inc.) equipped with QCapture Pro software (Q Imaging, Surrey, BC, Canada).

### Toxins.

TSST-1 was purified from S. aureus stationary-phase media in its native form through combinations of ethanol precipitation and thin-layer isoelectric focusing (60) or 6×His tagged using Ni^2+^ affinity chromatography, as described below. Both preparations resulted in a single band by Coomassie blue stain. All TSST-1 and TSST-1 toxoid preparations (native or His tagged) were passed through a Detoxi-Gel LPS-removing resin (Thermo Scientific) to reduce LPS contamination to <0.1 ng of LPS per 3 mg of toxin (0.0008 ng/ml of LPS in 25 μg/ml of TSST-1), as tested by Limulus amebocyte lysate assay for LPS. LPS O113:H10 (Associates of Cape Cod, Inc., East Falmouth, MA) was reconstituted with LAL Water (Lonza, Walkersville, MD) and diluted in complete endothelial cell medium to a working concentration.

### Construction and expression of TSST-1 toxoid G31S/S32P/H135A.

The TSST-1 toxoid is a superantigenicity null molecule, in which both the MHC-II and TCR binding sites are inactivated (42, 44, 61). The MHC-II binding site mutation (G31S/S32P) and the TCR binding site mutation (H135A) have been well characterized both structurally and functionally and used successfully as an experimental vaccine component in rabbit models of S. aureus endocarditis, pneumonia, and sepsis (62, 63). The pCE104 shuttle vector encoding TSST-1_G31S/S32P_ (pCE107) was used as a template in a QuikChange mutagenesis reaction using tsthH135Afor and tsthH135Arev primers to introduce the H135A mutation. The resulting plasmid was maintained in DH5α and sequence verified. The gene encoding TSST-1_G31S/S32P/H135A_ or wild-type TSST-1 was PCR amplified using the tsthNdeF and tsthpET2bXhoIR primers and ligated into a pET25b expression vector. Plasmids encoding His-tagged TSST-1 or TSST-1 toxoid were introduced into Escherichia coli BL21(DE3) and grown at 37°C with shaking to an optical density at 600 nm (OD_600_) of 0.5. Strains were maintained in LB supplemented with carbenicillin (100 μg/ml). Protein expression was induced with isopropyl-β-d-thiogalactopyranoside (IPTG; 1 mM) for 2 to 4 h at 30°C. Bacterial pellets were resuspended in imidazole buffer (20 mM Tris-HCl [pH 8.0], 250 mM NaCl, 20 mM imidazole) supplemented with two protease inhibitor cocktail tablets (Roche) and lysed using a Microfluidizer (Microfluidics, Newton, MA). Protein was purified using Ni^2+^ affinity chromatography, dialyzed overnight in phosphate-buffered saline (PBS) at 4°C, and passed through a Detoxi-Gel endotoxin-removing resin (Thermo Scientific). Primers used included tsthH135Afor (ATTCGTGCACAGCTAACTCAAATACATGGATTATATCGT), tsthH135A rev (TTGAGTTAGCTGTGCACGAATTTCAAAGTCTAA), tsthNdeF (TTACCATATGTCTACAAACGATAATATAAAGG), and tsthpET2bXhoIR (TTACCTCGAGATTAATTTCTGCTTCTATAGTTT).

### MHC-II blocking.

For MHC-II receptor blocking on iHAECs, we used TÜ39 monoclonal antibody, which specifically recognizes human MHC-II HLA-DR, DP, and most DQ antigens; an isotype control antibody, purified mouse IgG2a, κ isotype, was used as a control (BD Biosciences, San Jose, CA). The effect of antibody pretreatment on iHAEC IL-8 secretion after TSST-1 stimulation was tested at concentrations between 0.05 and 50 μg/ml, and the testing yielded consistent results. Blocking and isotype control antibody at a concentration of 10 μg/ml was used for iHAEC pretreatment for 30 min prior to addition of TSST-1 with or without LPS.

### MTS assay.

Metabolic activity as a proxy measure of viability of the cells was examined by the CellTiter 96 AQ_ueous_ One Solution cell proliferation assay (Promega Corporation, Madison, WI) according to the manufacturer's instructions. iHAECs were plated into the 1% gelatin-coated 96-well plates (7,000 cells/well), grown to ∼90% confluence, and stimulated with toxin(s) in triplicates for 24 h. Twenty microliters of MTS solution [3-(4,5-dimethylthiazol-2-yl)-5-(3-carboxymethoxyphenyl)-2-(4-sulfophenyl)-2H-tetrazolium] was added to wells containing treated cells in 100 μl of medium 200 with LSGS/well. The plate was gently tapped to mix the contents and incubated for 2 h, and absorbance was read on a Tecan Infinite M200 plate reader (Tecan Group Ltd., Zürich, Switzerland). Data are reported as means for triplicate samples ± standard errors of the means (SEM).

### In-Cell Western assay.

The iHAECs (7,000 cells/well) were grown to near confluence in 1% gelatin-coated black optical tissue culture-treated 96-well plates (Nunc, Thermo Scientific, Waltham, MA) and stimulated with toxins for 24 h. An In-Cell Western assay was performed according to the manufacturer's instructions (LI-COR, Lincoln, NE). Briefly, the cells in plates were fixed with 3.7% paraformaldehyde, washed with PBS, permeabilized with 0.2% Tween 20, blocked with Odyssey blocking buffer (LI-COR) and 10% goat serum, and incubated with primary antibodies at 4°C overnight (O/N), secondary antibodies for 1 h at room temperature, and DRAQ5 (a nuclear stain used for normalization of target signal to total cell number per well) for 30 min, with extensive washing between the staining steps. Primary antibodies were the same as those used for flow cytometry; the secondary antibody was the corresponding goat anti-mouse or anti-rabbit antibody IRDye 800CW infrared dye (LI-COR). The LI-COR Odyssey CLx infrared imaging system was used to capture digital images of the cells in plates, and the fluorescence intensity of IRDye and DRAQ5 was quantified with ImageJ. The fluorescence of the antibody was normalized to the number of cells in each well identified with a DRAQ5 nuclear stain. Data are expressed as a fluorescence intensity of toxin-treated cells relative to that of cells in medium wells. Controls consisted of the cells incubated with secondary antibodies alone and cells with no antibodies; the background fluorescence of these control cells was negligible.

### Endothelial-cell monolayer permeability assay.

iHAECs were seeded onto a 0.2% gelatin-coated 24-well plate (40,000 cells/well) with 0.4-μm-pore-size Transwell membranes in 100 μl of medium, with bottom wells filled with 600 μl of medium, and grown until confluence (confirmed by phase-contrast microscopy). Media in both top and bottom compartments were exchanged for media with or without LPS (1 ng/ml), TSST-1 (12.5 μg/ml), or both toxins together, and plates were incubated for 24 h. The next day, the top wells were spiked with Lucifer yellow or BSA-Alexa Fluor 633 for a final concentration of 20 μg/ml each. Positive control wells were preincubated with EDTA 30 min prior to addition of the tracers. An aliquot of 50 μl was removed from the bottom wells at 10, 20, 30, 40, and 120 min and placed into a 96-well black plate with optical bottoms, and fluorescence was read on a Tecan Infinite M200 plate reader.

### Wound healing assay.

To examine the effects of toxins on iHAEC migration and proliferation *in vitro*, we used a wound healing assay. iHAECs (40,000/well) were grown until near confluence in 1% gelatin-coated 24-well plates, and after O/N stimulation with toxins, a scratch in the cellular monolayers was made with a sterile pipette tip. Phase-contrast images of the scratches were taken at time zero and at various time points thereafter. Quantification of a scratched area that remained open was performed with ImageJ and is reported as a ratio of an area of scratch at 19 and 0 h postscratch. The 19-h postscratch time was chosen for data analysis, as we aimed to capture the monolayers before a complete closure of the wound. Each experimental group consisted of 3 or more wells, and results from a representative of 4 independent experiments yielding consistent results are shown.

### IL-8 and IL-6 ELISAs.

pHAECs or iHAECs were plated into 1% gelatin-coated 96-well plates (7,000 cells/well) or 24-well plates (40,000 cells/well), grown until near confluence, and incubated with medium, LPS, TSST-1, or LPS plus TSST-1 in triplicates. After 22 to 24 h, the cell culture medium was collected and aliquots were frozen for later analysis. Chemokine IL-8 and cytokine IL-6 in the culture supernatants were assayed by ELISA (BD Biosciences, San Jose, CA) according to the manufacturer's instructions. Briefly, a 96-well Nunc-MaxiSorp plate was coated O/N with a capture antibody, washed and blocked with assay diluent (PBS with 10% fetal bovine serum). Samples were diluted in an assay diluent, incubated in plates O/N, and developed with a secondary antibody and colorimetric substrate, and absorbance was read at 450 nm with 570-nm wavelength correction on a Tecan Infinite M200 microplate reader (Tecan Group Ltd.). Concentrations of IL-6 and IL-8 in samples were determined against the standard curve generated using serial dilutions of provided standard cytokine/chemokine with known concentrations. Data are reported as means from triplicate samples ± SEM.

### Statistical analyses.

Statistical significance across means was carried out using one-way or two-way analysis of variance (ANOVA) (as specified in the figure legends), with Holm-Sidak's multiple comparisons for differences between the groups (GraphPad Prism software). For clarity, the adjusted *P* value is reported for the conditions relevant to the question being asked (for example, TSST-1 versus medium or versus LPS plus TSST-1 or LPS versus LPS plus TSST-1). A *P* value equal to or smaller than 0.05 is considered significant.

## Supplementary Material

Supplemental material
