# Supplementary material for: The Superantigen Toxic Shock Syndrome Toxin 1 Alters Human Aortic Endothelial Cell Function
Source: Infect Immun. 2018 Feb 20;86(3):e00848-17. doi: 10.1128/IAI.00848-17 (PMC5820935; doi:10.1128/IAI.00848-17)
Supplement: Supplemental material [file supp_86_3_e00848-17__index.html]

Supplemental material 

# The Superantigen Toxic Shock Syndrome Toxin 1 Alters Human Aortic Endothelial Cell Function

## Supplemental material

- Supplemental file 1 -

  Fig. S1 to S5 legends.

  PDF, 80K
- Supplemental file 2 -

  Fig. S1. Protein expression levels of endothelial cell markers and receptors important in HAEC activation and amplification of immune responses.

  PDF, 1.4M
- Supplemental file 3 -

  Fig. S2. TSST-1-treated iHAECs display a discontinuous VE-cadherin membrane staining pattern.

  PDF, 5.6M
- Supplemental file 4 -

  Fig. S3. iHAECs metabolic activity and IL-8 secretion under experimental conditions.

  PDF, 103K
- Supplemental file 5 -

  Fig. S4. TSST-1 suppresses secretion of IL-8 and IL-6 in LPS-stimulated primary HAECs.

  PDF, 168K
- Supplemental file 6 -

  Fig. S5. TSST-1 suppresses IL-8 responses to LPS in iHAECs.

  PDF, 96K
