# Supplementary material for: The Superantigen Toxic Shock Syndrome Toxin 1 Alters Human Aortic Endothelial Cell Function
Source: Infect Immun. 2018 Feb 20;86(3):e00848-17. doi: 10.1128/IAI.00848-17 (PMC5820935; doi:10.1128/IAI.00848-17)
Supplement: Supplemental material [file IAI.00848-17_zii999092311s1.pdf]

**SUPPLEMENTAL MATERIAL FOR PUBLICATION**

**SUPPLEMENTARY FIGURE LEGENDS**

**FIGURE S1. Protein expression levels of endothelial cell markers (A) and receptors important in HAEC activation and amplification of immune responses (B).** Flow cytometry histogram plots of non-stimulated iHAECs labeled with (A) vWF (von Willebrand factor)-AF647 or eNOS (endothelial nitric oxide synthase)-AF647, and (B) MHCII-AF488, TLR4-AF647, CD40-AF488, or CD14-AF647 antibodies. Secondary antibody only-stained cells (dashed line) and non-stained cells (dotted line) were used as controls.

**FIGURE S2. TSST-1 treated iHAECs display a discontinuous VE-cadherin membrane staining pattern.** iHAECs were grown to confluence on gelatin-coated coverslips and left untreated (A) or treated with TSST-1 at 10 µg/ml (B) or 100 µg/ml (C) for 24 h, fixed and stained with anti-VE-cadherin antibody (green). Nuclei were counter-stained with DAPI (blue). Slides were imaged on an epifluorescent microscope (left panels). Regions from the center of the field on the left panel (dotted areas) were expanded in the right panels to show detail. Arrows indicate areas of discontinuous VE-cadherin staining pattern at the cell-cell contact interfaces. Also visualizes a decrease in overall VE-cadherin staining intensity in TSST-1 treated cells compared to not-treated cells (media).

**FIGURE S3. iHAECs metabolic activity and IL-8 secretion in experimental conditions.** (A) Metabolic activity determined by the MTS assay in iHAECs stimulated with TSST-1 native, TSST-1 His-tagged (control), or TSST-1 Toxoid His-tagged at 25 µg/ml with or without LPS at

1 ng/ml. (B) Metabolic activity determined by the MTS assay in iHACEs stimulated with increasing concentrations of TSST-1 native, TSST-1 His-tagged (control), or TSST-1 Toxoid His-tagged (μg/ml). (C) iHAECs stimulated with increasing concentrations of TSST-1 native, TSST-1 His-tagged, or TSST-1 Toxoid His-tagged (μg/ml), and with LPS (ng/ml) as a positive control. IL-8 secreted into culture supernatants was measured by ELISA. Statistics by one-way ANOVA with Holm-Sidak's multiple comparisons: (A) ns = not significant; (B) **$p<0.01$. (C) **** $p<0.0001$.

**FIGURE S4. TSST-1 suppresses secretion of IL-8 and IL-6 in LPS-stimulated primary HAECs.** (A) Primary HAECs stimulated with increasing concentrations of LPS (0.005 – 2.5 ng/ml) in the presence of TSST-1 (3 μg/ml for IL-8; and 25 μg/ml for IL-6) for 24 h. IL-8 or IL-6 were measured by ELISA. P-values determined by two-way ANOVA (stars). ***$p=0.0001$ (LPS vs LPS + TSST-1 across all concentrations). (B) Percent suppression on LPS-mediated IL-8 or IL-6 secretion resulting from concomitant stimulation with TSST-1. P-values determined by one-way ANOVA (****$p<0.0001$).

**FIGURE S5. TSST-1 suppresses IL-8 responses to LPS in iHAECs.** iHAECs pre-treated for 4 h with TSST-1 (12.5 μg/ml) or LPS (0.5 ng/ml) before addition of the other co-stimulant: LPS (0.5 ng/ml) or TSST-1 (12.5 μg/ml), respectively, for an additional 24 h. P-values determined by one-way ANOVA (excluding media and TSST-1) with Holm-Sidak's multiple comparisons test (stars, adjusted P-value). ***$p=0.0003$ (no pre-stimulation); ***$p=0.0004$ (pre-stimulation).
