# Supplementary material for: The Superantigen Toxic Shock Syndrome Toxin 1 Alters Human Aortic Endothelial Cell Function
Source: Infect Immun. 2018 Feb 20;86(3):e00848-17. doi: 10.1128/IAI.00848-17 (PMC5820935; doi:10.1128/IAI.00848-17)
Supplement: Supplemental material [file IAI.00848-17_zii999092311s2.pdf]

Figure S1.

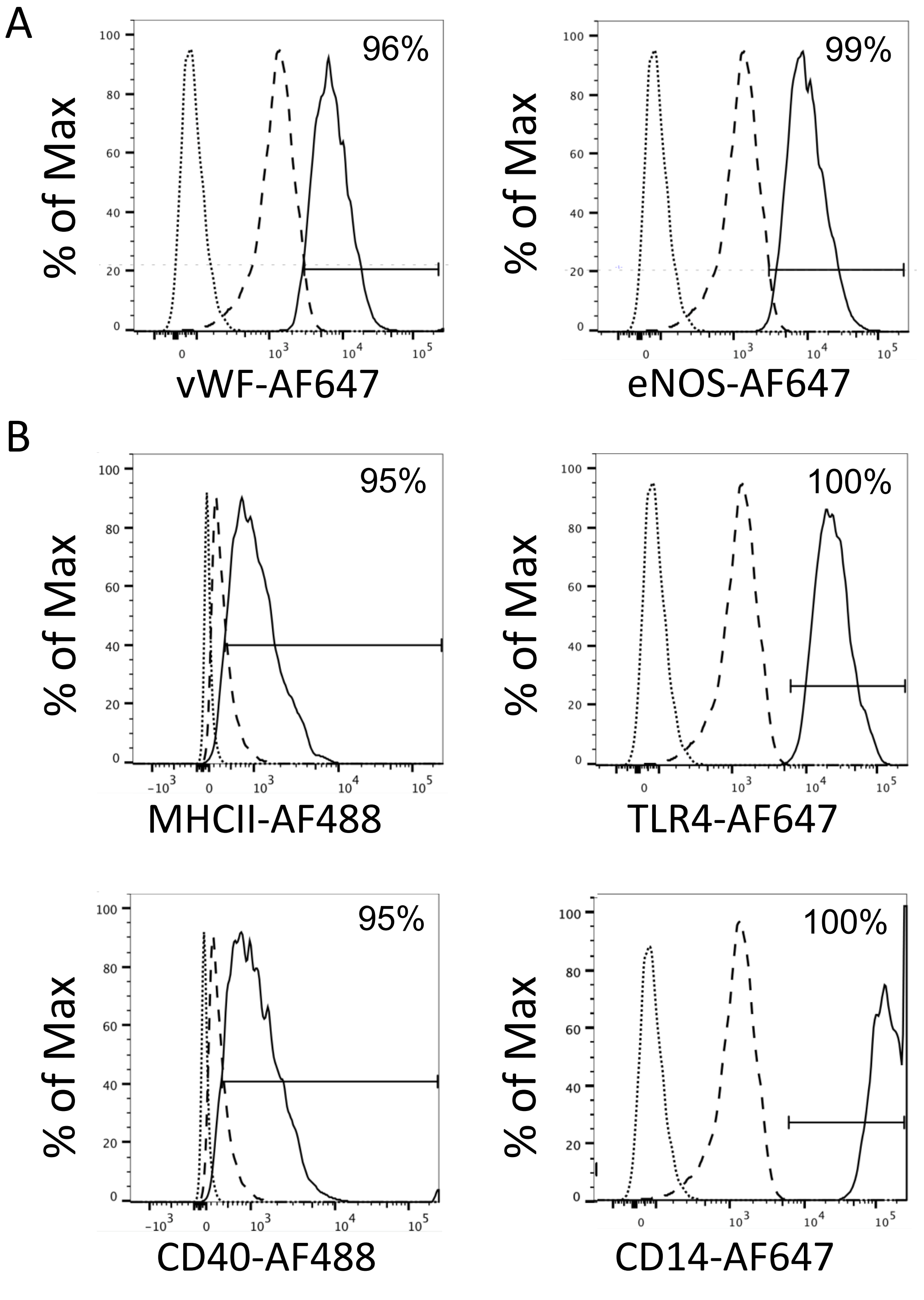

FIG S1. Protein expression levels of endothelial cell markers (A) and receptors important in HAEC activation and amplification of immune responses (B). Flow cytometry histogram plots of non-stimulated iHAECs labeled with_(A) vWF (von Willebrand factor)-AF647 or eNOS (endothelial nitric oxide synthase)-AF647, and (B) MHCII-AF488, TLR4-AF647, CD40-AF488, or CD14-AF647 antibodies (solid line). Secondary antibody only-stained cells (dashed line) and non-stained cells (dotted line) were used as controls.
