# Supplementary material for: The Superantigen Toxic Shock Syndrome Toxin 1 Alters Human Aortic Endothelial Cell Function
Source: Infect Immun. 2018 Feb 20;86(3):e00848-17. doi: 10.1128/IAI.00848-17 (PMC5820935; doi:10.1128/IAI.00848-17)
Supplement: Supplemental material [file IAI.00848-17_zii999092311s3.pdf]

Figure S2.

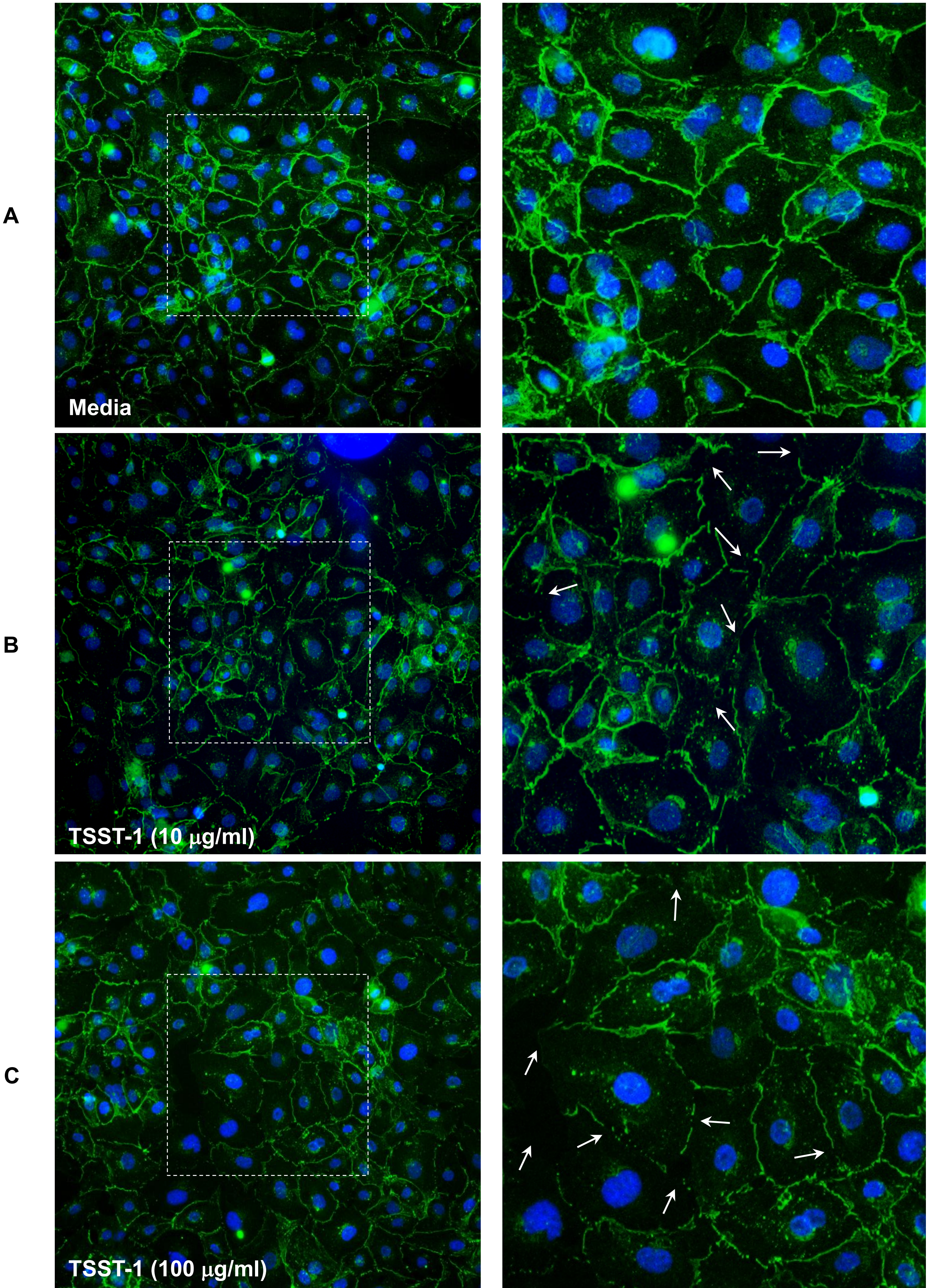

FIG S2. TSST-1 treated iHAECs display a discontinuous VE-cadherin membrane staining pattern. iHAECs were grown to confluence on gelatin-coated coverslips and left untreated (A) or treated with TSST-1 at 10 μg/ml (B) or 100 μg/ml (C) for 24 h, fixed and stained with anti-VE-cadherin antibody (green). Nuclei were counter-stained with DAPI (blue). Slides were imaged on an epifluorescent microscope (left panels). Regions from the center of the field on the left panel (dotted areas) were expanded in the right panels to show detail. Arrows indicate areas of discontinuous VE-cadherin staining pattern at the cell-cell contact interfaces. Also visualizes a decrease in overall VE-cadherin staining intensity in TSST-1 treated cells compared to not-treated cells (media).
