# Supplementary material for: The Superantigen Toxic Shock Syndrome Toxin 1 Alters Human Aortic Endothelial Cell Function
Source: Infect Immun. 2018 Feb 20;86(3):e00848-17. doi: 10.1128/IAI.00848-17 (PMC5820935; doi:10.1128/IAI.00848-17)
Supplement: Supplemental material [file IAI.00848-17_zii999092311s4.pdf]

Figure S3.

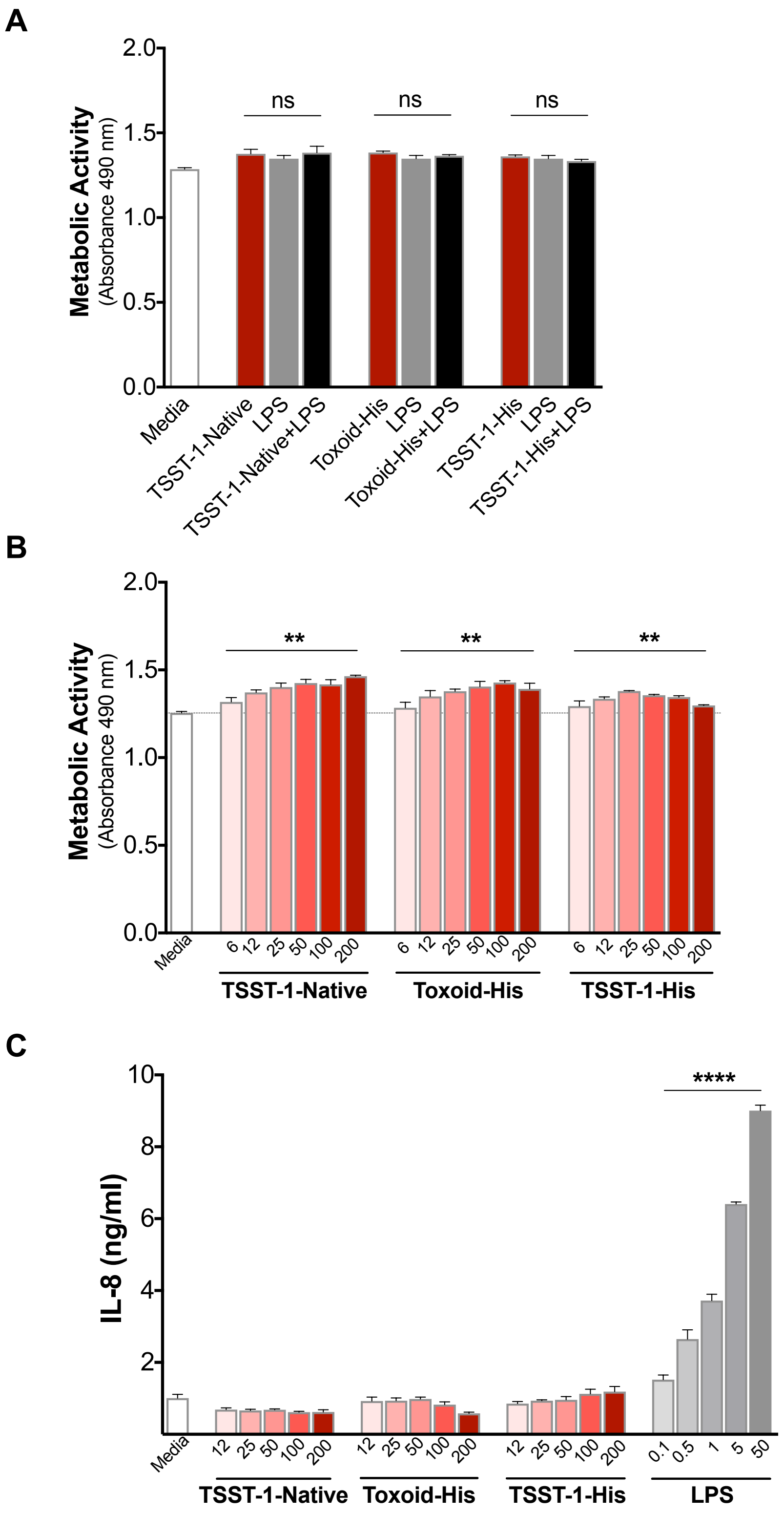

FIG S3. iHAECs metabolic activity and IL-8 secretion in experimental conditions. (A) Metabolic activity determined by the MTS assay in iHAECs stimulated with TSST-1 native, TSST-1 His-tagged (control), or TSST-1 Toxoid His-tagged at 25 μg/ml with or without LPS at 1 ng/ml. (B) Metabolic activity determined by the MTS assay in iHACEs stimulated with increasing concentrations of TSST-1 native, TSST-1 His-tagged (control), or TSST-1 Toxoid His-tagged (μg/ml). (C) iHAECs stimulated with increasing concentrations of TSST-1 native, TSST-1 His-tagged, or TSST-1 Toxoid His-tagged (μg/ml), and with LPS (ng/ml) as a positive control. IL-8 secreted into culture supernatants was measured by ELISA. Statistics by one-way ANOVA with Holm-Sidak's multiple comparisons: (A) ns = not significant; (B) **$p<0.01$. (C) **** $p<0.0001$.
