# Supplementary material for: The Superantigen Toxic Shock Syndrome Toxin 1 Alters Human Aortic Endothelial Cell Function
Source: Infect Immun. 2018 Feb 20;86(3):e00848-17. doi: 10.1128/IAI.00848-17 (PMC5820935; doi:10.1128/IAI.00848-17)
Supplement: Supplemental material [file IAI.00848-17_zii999092311s5.pdf]

Figure S4.

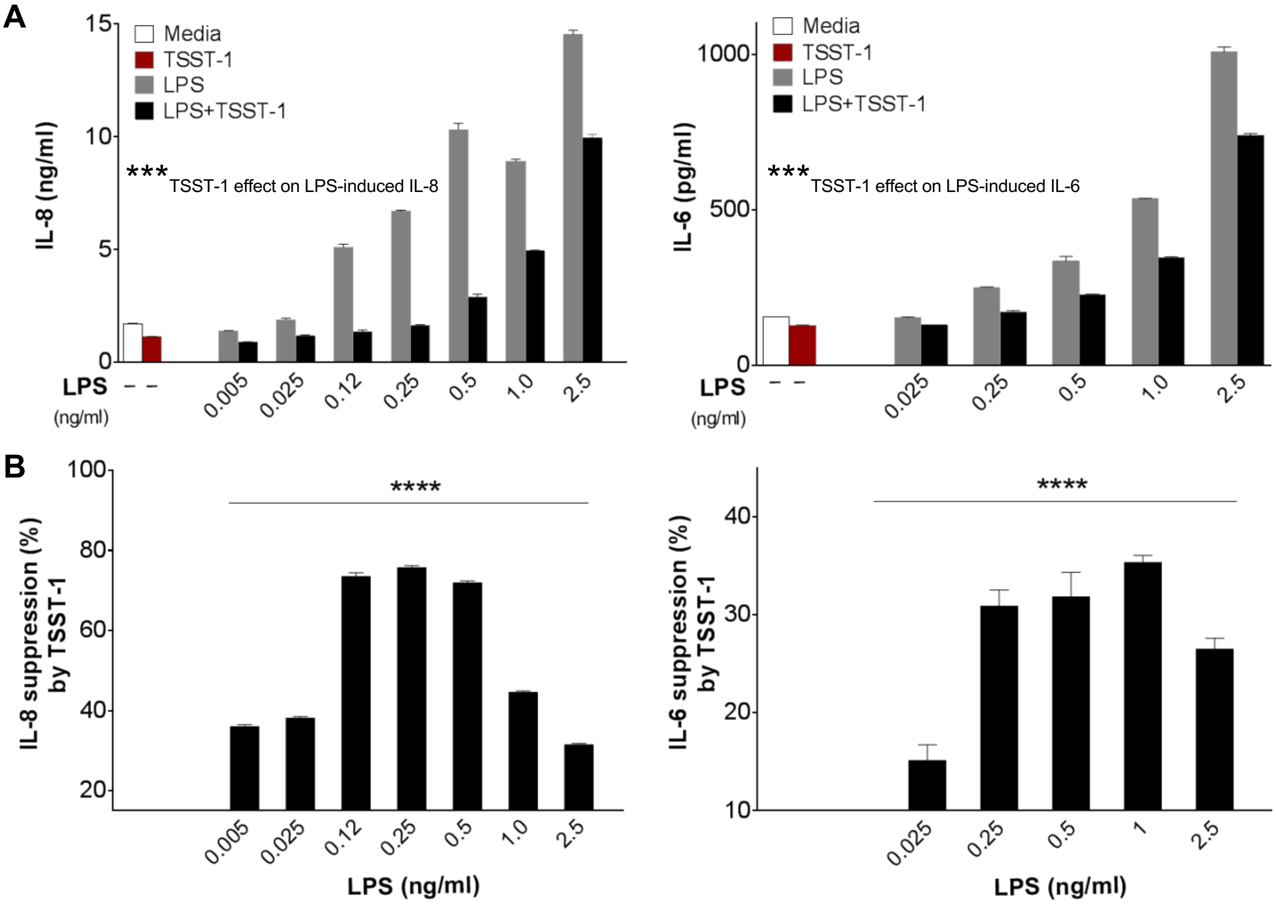

FIG S4. TSST-1 suppresses secretion of IL-8 and IL-6 in LPS-stimulated primary HAECs. (A) Primary HAECs stimulated with increasing concentrations of LPS (0.005 – 2.5 ng/ml) in the presence of TSST-1 (3 µg/ml for IL-8; and 25 ug/ml for IL-6) for 24 h. IL-8 or IL-6 were measured by ELISA. P-values determined by two-way ANOVA (stars). ****p*=0.0001 (LPS vs LPS + TSST-1 across all concentrations). (B) Percent suppression on LPS-mediated IL-8 or IL-6 secretion resulting from concomitant stimulation with TSST-1. P-values determined by one-way ANOVA (*****p*<0.0001).
