# Supplementary material for: The Superantigen Toxic Shock Syndrome Toxin 1 Alters Human Aortic Endothelial Cell Function
Source: Infect Immun. 2018 Feb 20;86(3):e00848-17. doi: 10.1128/IAI.00848-17 (PMC5820935; doi:10.1128/IAI.00848-17)
Supplement: Supplemental material [file IAI.00848-17_zii999092311s6.pdf]

Figure S5.

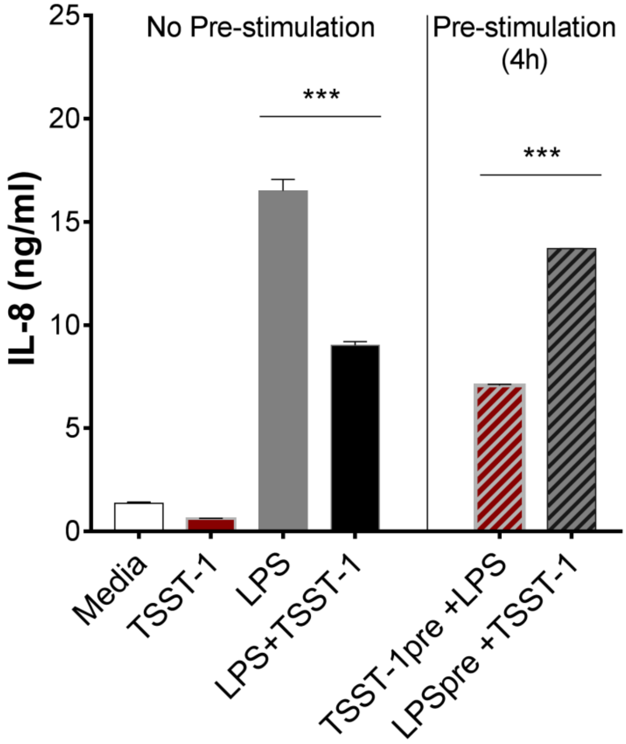

FIG S5. TSST-1 suppresses IL-8 responses to LPS in iHAECs. iHAECs pre-treated for 4 h with TSST-1 (12.5 μg/ml) or LPS (0.5 ng/ml) before addition of the other co-stimulant: LPS (0.5 ng/ml) or TSST-1 (12.5 μg/ml), respectively, for an additional 24 h. P-values determined by one-way ANOVA (excluding media and TSST-1) with Holm-Sidak's multiple comparisons test (stars, adjusted P-value). ****p*=0.0003 (no pre-stimulation); ****p*=0.0004 (pre-stimulation).
